# Design and engineering of bispecific antibodies: insights and practical considerations

**DOI:** 10.3389/fbioe.2024.1352014

**Published:** 2024-01-25

**Authors:** Andreas V. Madsen, Lasse E. Pedersen, Peter Kristensen, Steffen Goletz

**Affiliations:** ^1^ Department of Biotechnology and Biomedicine, Technical University of Denmark, Kongens Lyngby, Denmark; ^2^ Department of Chemistry and Bioscience, Aalborg University, Aalborg, Denmark

**Keywords:** bispecific, antibody, immunoglobulin, IgG fusion, sdAb, scFv, HC heterodimerization

## Abstract

Bispecific antibodies (bsAbs) have attracted significant attention due to their dual binding activity, which permits simultaneous targeting of antigens and synergistic binding effects beyond what can be obtained even with combinations of conventional monospecific antibodies. Despite the tremendous therapeutic potential, the design and construction of bsAbs are often hampered by practical issues arising from the increased structural complexity as compared to conventional monospecific antibodies. The issues are diverse in nature, spanning from decreased biophysical stability from fusion of exogenous antigen-binding domains to antibody chain mispairing leading to formation of antibody-related impurities that are very difficult to remove. The added complexity requires judicious design considerations as well as extensive molecular engineering to ensure formation of high quality bsAbs with the intended mode of action and favorable drug-like qualities. In this review, we highlight and summarize some of the key considerations in design of bsAbs as well as state-of-the-art engineering principles that can be applied in efficient construction of bsAbs with diverse molecular formats.

## 1 Introduction

In recent years, bispecific antibodies (bsAbs) have emerged as a powerful class of therapeutic molecules, opening new avenues for targeted therapy. BsAbs possess the unique ability to simultaneously engage two different targets, enabling synergistic targeting of disease pathways that cannot be obtained even with combinations of conventional immunoglobulins. While therapeutic bsAbs are engineered molecules, bsAbs can also be found in rare cases in nature where they can be formed through Fab-arm exchange of IgG4 half molecules (Rispens et al., 2011). The binding versatility of engineered bsAbs has sparked significant interest in their potential applications across a wide range of diseases, including cancer, autoimmune disorders, and infectious diseases (Labrijn et al., 2019). In a recent publication, bi- and multispecific drugs were even classified as a new wave of transformative therapeutics (Deshaies, 2020). The therapeutic potential of bsAbs has made the bsAb space very crowded and extremely competitive but it has also fostered truly ingenious feats of engineering as illustrated by the diversity of the clinical-stage bsAb landscape (Labrijn et al., 2019; Wei et al., 2022).

## 2 Mechanisms of action

The dual binding activity of bsAbs enables synergistic antigen targeting with more complex mechanisms of action (MOA) than what can be obtained for conventional monoclonal antibodies. For many bsAbs the desired MOA is dependent on a certain spatiotemporal connection between the two binding events. This means that the two binding specificities must be engaged in a specific physical arrangement (e.g., positioning targets near each other to induce downstream signaling (Sampei et al., 2013)) and/or with a specific timing (e.g., simultaneously linking cells (Li et al., 2021) or sequential targeting for translocating across barriers (Wec et al., 2016; Do et al., 2020)). Such bsAbs where the MOA cannot be obtained through combination of the two separate parental antibodies are known as obligate bsAbs (Spiess et al., 2015; Labrijn et al., 2019). However, even bsAbs without obligate MOA often corroborate to be more than simply the sum of their parts, which is illustrated by non-obligate bsAbs still showing superior potency relative to combination of the parental antibodies (Kast et al., 2021). The advantage of such combinatorial MOA has been speculated to stem from avidity effects (De Gasparo et al., 2021). Dual targeting might also help minimize side effects by improving target selectivity and localization (Mazor et al., 2015a; Mazor et al., 2017), which is especially relevant to limit “on-target, off-tumor” effects (Ishiguro et al., 2017). Even in cases where the bsAb activity is comparable to that of the parental antibody combination, the bsAb may offer an advantage regarding manufacturing since only a single molecule needs to be produced (Hmila et al., 2010).

The dual targeting activity of bsAbs can broadly be characterized as working *in-trans* or *in-cis*. The possibility of judiciously combining selected antigen-binding domains to specifically act on the same (*in-cis*) or different (*in-trans*) cellular/molecular targets in a highly tailored manner is essential in synergistic bsAb functionalities.

**FIGURE 1:**
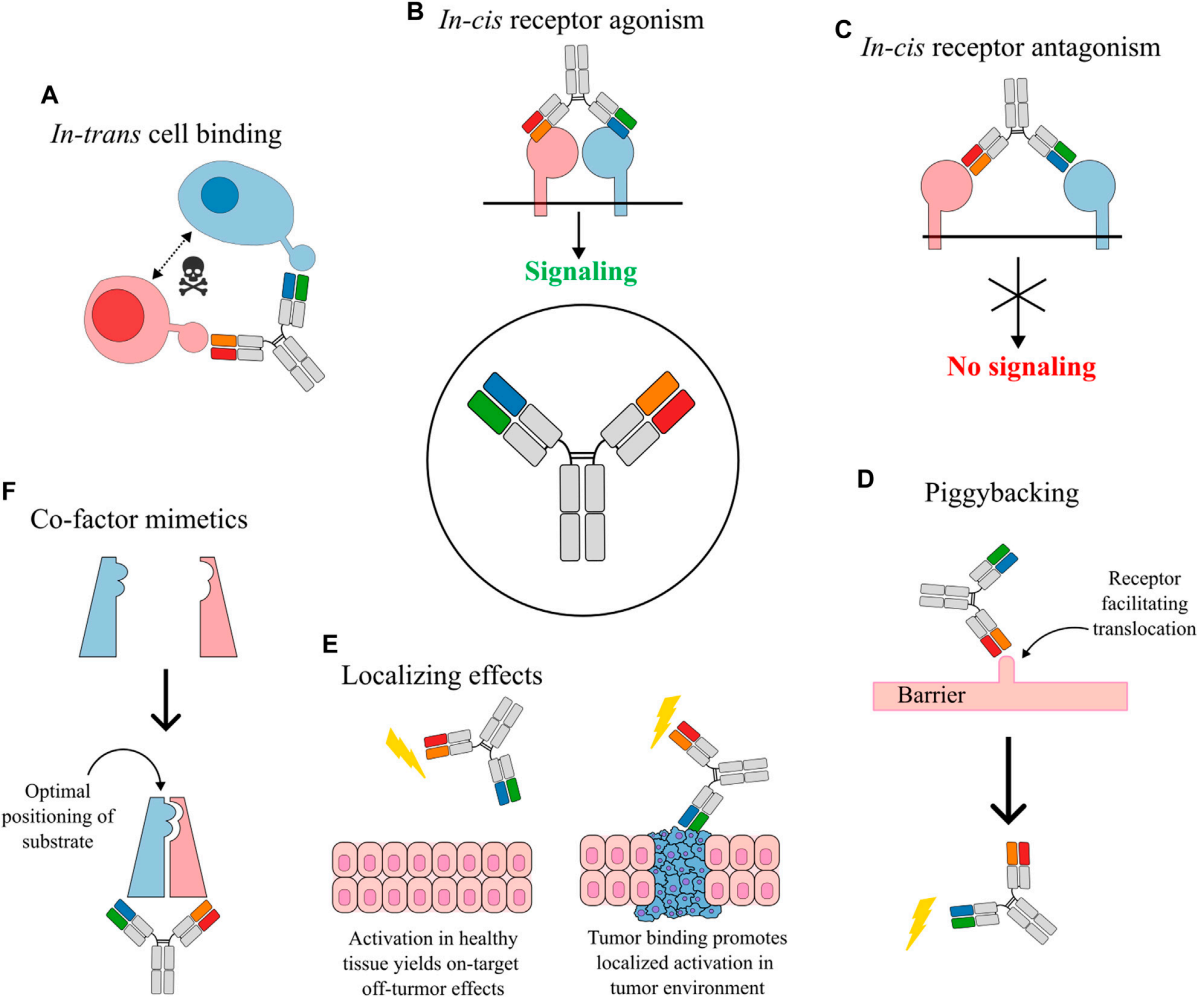
Obligate mechanisms of action for bsAbs. **(A)** *In-trans* cell bridging established a physical link between different cells through the bsAb. This mechanism is especially relevant for T cell redirecting bsAbs where the physical connection helps target the cytotoxicity of the activated T cells. **(B)** *In-cis* bridging of receptors causes agonistic crosslinking and associated activation of receptor signaling. **(C)** *In-cis* antagonism blocks receptor association thus preventing signaling through the receptor complex. **(D)** Piggybacking bsAbs use one specificity for targeting a receptor that facilitates translocation across a barrier to an otherwise inaccessible compartment where the second specificity exerts its functionality. The lightning bolt indicates an activity mediated by binding of the antibody. **(E)** BsAbs can exert localization effects. An example is agonistic bsAbs that also contain a tumor-targeted specificity, which limits on-target, off-tumor effects by restricting the agonistic mechanism to sites where the tumor marker is present. **(F)** BsAbs acting as a co-factor mimetic by accurately positioning of enzyme and substrate.

*In-trans* acting bsAbs engage distinct targets to create a physical linkage. For bsAbs engaging cellular targets *in-trans* this means the MOA is dependent on binding of two different cells thus permitting bridging of the cells. A common example of *in-trans* cellular binding are bispecific T cell engagers that bind to both a tumor associated antigen and CD3, a highly potent costimulatory receptor, thus bridging T cell and tumor cells (Löffler et al., 2000; Hoffmann et al., 2005) (Figure 1A). The bispecific T cell engagers are bypassing the natural T cell activation through clustering of low-affinity T cell receptors (Krogsgaard and Davis, 2005) and recruit T cells directly towards cells expressing the tumor-associated antigen (Löffler et al., 2000). The term “*in-trans*” has also been used on a molecular level to describe a biparatopic anti-HER2 bsAb that bind two distinct HER2 molecules (*in-trans*) rather than the same (*in-cis*). The cross-binding links adjacent molecules to induce distinct HER2 reorganization with complement-dependent cytotoxicity not seen for combinations of monospecific anti-HER2 antibodies (Weisser et al., 2023). Such molecular *in-trans* binding is primarily relevant for biparatopic bsAbs targeting different and non-overlapping epitopes on the same antigenic target.

*In-cis* acting bsAbs primarily target antigens on the same cell (Figures 1B,C). Agonistic bsAbs that activate two receptors on the surface of the same cell (cellular *in-cis* activation) can be used to selectively control signaling mechanisms by precisely steering bridging of receptor subunits. In one example, a bsAb targeting the interleukin (IL)-2 receptor subunits IL-2Rβ and IL-2Rγ was used to increase signaling through the intermediate affinity IL-2Rβγ while downregulating signaling through the high affinity IL-2Rαβγ (Ha et al., 2021). In another example a dual agonistic bispecific Surrobody targeting death receptor (DR) 4 and 5 showed superior potency than combinations of the parental antibodies potentially due to heterodimeric clustering of DR4 and DR5 (Milutinovic et al., 2016).

Another type of *in-cis* activating bsAbs is known as co-factor mimetics that essentially acts to promote optimal positioning of an enzyme and its cognate substrate (Figure 1F). Coagulation factor FVIIIa mimetics have been developed to promote complexation of FIXa and FX (Sampei et al., 2013; Østergaard et al., 2021). Assembly of the FIXa-FX enzyme-substrate complex leads to formation of activated factor Xa (FXa), which is a key mediator of the coagulation pathway (Borensztajn et al., 2008) and the bsAbs are therefore relevant for treatment of hemophilia A (Shima et al., 2016; Mahlangu et al., 2018; Lauritzen et al., 2022). This bsAb cofactor mimetic is especially relevant for patients that develop alloantibodies against FVIII and which are therefore largely restricted from treatment with recombinant FVIII (Muto et al., 2014).

Signaling pathways can also be targeted through antagonistic bsAbs working by occupying cellular receptors and blocking binding of soluble receptor ligands. Such antagonistic bsAbs are commonly used in blocking of immune checkpoint receptors, which is an extremely successful class of anti-cancer immunotherapy targets (Robert, 2020). Examples include dual blockade of immune checkpoint pathways (Kraman et al., 2020; Chen et al., 2021; Dovedi et al., 2021) and targeting of immune checkpoints together with negative regulators of anti-tumor immunity, such as TGF-β, that is associated with poor prognosis (Bai et al., 2019; Yi et al., 2021). Antagonistic bsAbs working *in-cis* can also be used for targeting related or overlapping signaling pathways (Dong et al., 2011). Such bsAbs can thus be used to preempt immune evasion of cancer cells by targeting a primary cancer-related pathway as well as other connected pathways that might be upregulated by the cancer cell in response to blocking of the primary pathway. An example of antagonistic dual pathway targeting is seen for HER2xHER3 bsAbs where upregulation of HER3 is a driver in resistance to HER2 inhibiting agents (Garrett et al., 2011).

Discovery of agonistic bsAbs is generally more challenging than antagonistic bsAbs because the agonistic activity of antibodies depends on multiple factors that are difficult to properly formalize (Mayes et al., 2018). As an example, the cytotoxic activity of agonistic anti-CD3 bsAbs have been found to be influenced by both the affinity, epitope, and molecular geometry (Bluemel et al., 2010; Moore et al., 2015; Root et al., 2016). Contrarily, an effective antagonistic antibody is typically characterized by a high affinity and ability to compete with the natural ligand thereby blocking its action. Further, additional synergies in therapeutic effector mechanisms might be obtained from combinations of bsAbs although this will likely increase the MOA complexity and thus further complicate bsAb design.

## 3 The role of Fc

All natural antibodies contain an Fc region that is paramount for the antibody effector functions, which triggers the host cell responses through binding to cognate Fc receptors and soluble immune mediators such as complement proteins (Lu et al., 2018). Fc protein engineering and glycoengineering can be used to either remove or largely enhance Fc-mediated effector functions such as antibody-dependent cellular cytotoxicity (ADCC) by NK cells, antibody-dependent cellular phagocytosis (ADCP) by macrophages and even certain CD8 T cell responses (Hart et al., 2017; Goletz et al., 2018; Liu et al., 2020). Significant efforts have been made to engineer Fc regions of conventional monospecific antibodies to obtain Fc effector functions (or lack thereof) that are not naturally observed for a given isotype (Saunders, 2019). As an example, FcγR silencing mutations can be used to create effector-reduced IgG1 antibodies that still possess the favorable characteristics that are associated with the IgG1 isotype (Tang et al., 2021; Cain et al., 2023). On the other hand, glycoengineering, such as de-core-fucosylation of Fc-IgG1 N-glycans, or mutations can increase binding to certain Fc receptors and thereby mediate increased effector functions such as FcγRIIIa mediated ADCC or ADCP. Such effector-modulating mutations or glycoengineering are typically not overlapping with common bsAb-related mutations (discussed below) such as those used for chain-steering and hence can be combined in Fc-containing bsAbs as well (Escobar-Cabrera et al., 2017). Depending on the intended MOA, certain bsAbs might be more effectively designed with a modified Fc region or without one altogether. Most clinically developed bsAbs contain a Fc region (Labrijn et al., 2019) as this domain confers easy purification using protein A (Liu et al., 2010) as well as prolonged half-life from FcRn recycling (Ghetie et al., 1996; Christianson et al., 1997). Fc-containing bsAbs are broadly categorized as symmetric or asymmetric according to their molecular geometry, as discussed below.

Despite effective engineering of the Fc region, there are cases where smaller fragment-based bsAbs offer advantages over large IgG-like bsAbs. Fragment-based bsAbs are typically constructed through genetic fusion of independent antigen-binding domains but can also be assembled through multimerization domains (Blanco-Toribio et al., 2013; Walter et al., 2022; Burke et al., 2023). This simplistic approach has the advantage that its products are typically small and without important glycosylation sites, meaning that they can be produced at high titers using simple expression systems like bacteria (Zhu et al., 1996; Lu et al., 2022) or yeast (Xiong et al., 2018). Fragment-based bsAbs typically consist of only one to two polypeptide chains, which limits the risk of antibody-related impurities due to chain mispairing, which is an issue often encountered for larger and structurally more complex bsAbs as discussed below. Further, simple genetic fusion of antigen-binding domains permits high flexibility with regard to combination of valencies and specificities as exemplified by 1 + 3 (Harwood et al., 2018) and 3 + 3 (Blanco-Toribio et al., 2013; Compte et al., 2018) bsAb formats.

**FIGURE 2:**
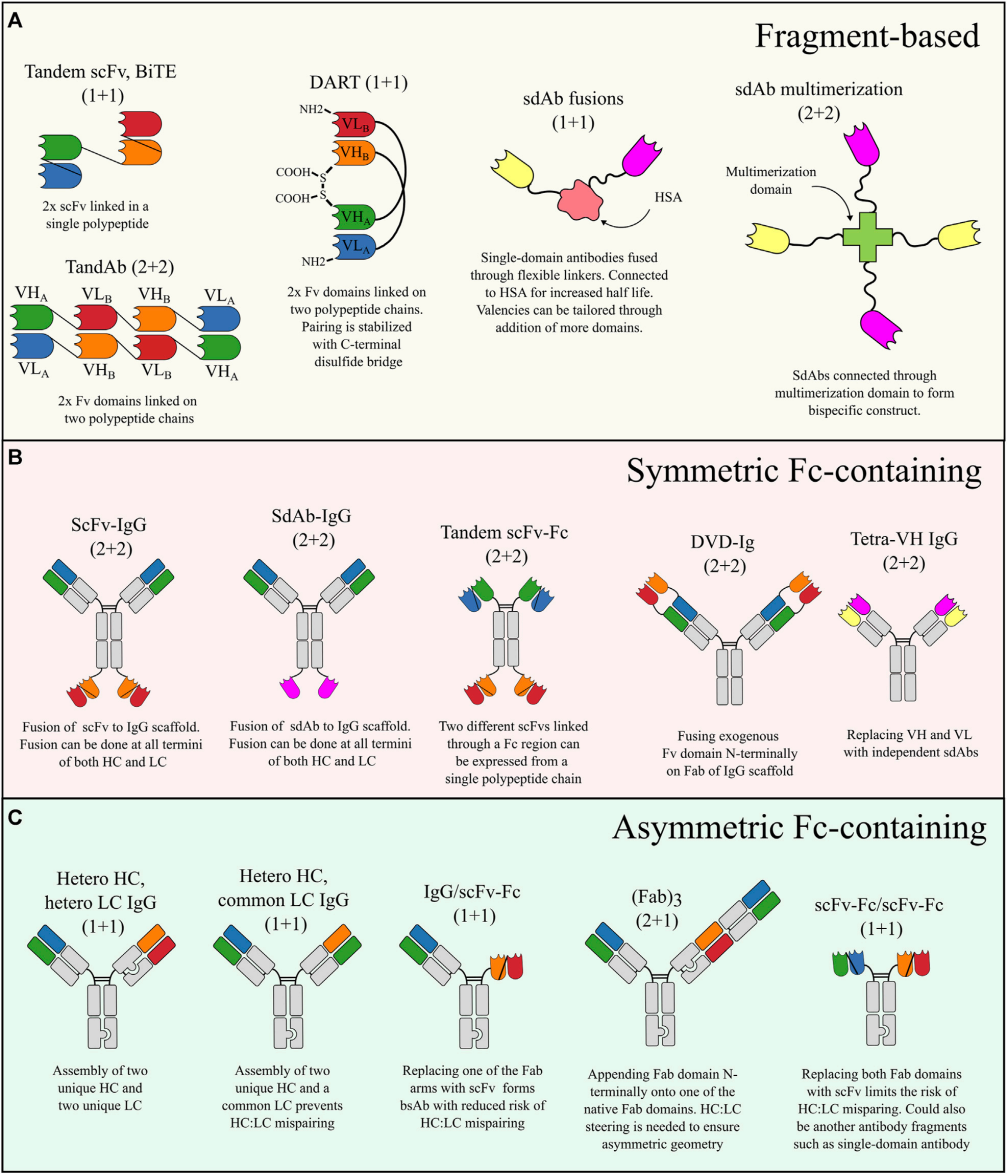
Schematic overview of the most common bsAb formats categorized according to molecular configuration. **(A)** Fragment-based bsAbs are formed by combining small antigen-binding fragments without a Fc domain. The building blocks are typically scFv fragments or sdAbs. **(B)** Symmetric Fc-containing bsAbs are expressed from one to two polypeptide chains often by fusing additional antigen-binding fragments onto an IgG scaffold. **(C)** Asymmetric Fc-containing bsAbs assembled from two to four polypeptide chains. Asymmetric bsAbs require measures to avoid chain mispairing. The small “knob” and “hole” in the schematics are used to generically illustrate places where a chain steering technology is needed to ensure HC:HC heterodimerization and/or proper HC:LC pairing.

Fragment-based bsAbs lacking an Fc region have traditionally been based on scFv fragments that can be combined in different ways to form, e.g., BiTE (Mack et al., 1995; Löffler et al., 2000), TandAb (Reusch et al., 2014; Reusch et al., 2015), or DART (Johnson et al., 2010; Chichili et al., 2015) molecules, which are the main classes of fragment-based bsAbs utilizing scFvs (Figure 2A). Such scFv fragments are, however, often associated with thermodynamic instability (Wörn and Plückthun, 2001) and promiscuous VH-VL pairing leading to undesired self-assembly (Weatherill et al., 2012). The limitations of scFvs have sparked interest in single-domain antibodies (sdAbs) which are also small (∼15 kDa) antigen-binding domains that can be genetically fused to form fragment-based bsAbs (Conrath et al., 2001; de Bruin et al., 2018) but because they are stable, robust, and monomeric in nature, they are not prone to undesired self-assembly. SdAbs have also been combined without linkers by fusion onto CH1 and Cκ, respectively, thus using the natural pairing of the constant domains as a natural dimerization motif to generate small Fab-like bsAbs (Rozan et al., 2013). It should of course be noted that antigen-binding domains can be combined in numerous other ways than those listed above, as previously described (Brinkmann and Kontermann, 2017). A potential drawback from fragment-based bsAbs is their faster clearance due to the small size and lack of FcRn binding regions, however, the clearance issue has been addressed for certain applications and formats by coupling of the bsAb to HSA (McDonagh et al., 2012; Mandrup et al., 2021; Hangiu et al., 2022) or including an anti-HSA antibody fragment (Austin et al., 2021; Edwards et al., 2022; Ishiwatari-Ogata et al., 2022).

## 4 Symmetric bispecifics

Symmetric bsAbs are typically IgG-like molecules that adhere to the HC_2_LC_2_ format but with additional exogenous binding domains fused to the scaffold thus adding specificity towards a second antigen to the original scaffold. These bsAbs are thus typically tetravalent 2 + 2 bispecifics (bivalent targeting of each antigen) (Pang et al., 2023) but can also be made as hexavalent 2 + 4 bsAbs by double fusions (Madsen et al., 2023). Some of the most common tetravalent 2 + 2 formats include scFv-IgG fusion (Orcutt et al., 2010; DiGiandomenico et al., 2014; Pang et al., 2023), DVD-Ig (Wu et al., 2007; Wu et al., 2009), tandem scFv-Fc (Chang et al., 2022), sdAb-IgG fusions (Madsen et al., 2022; Yanakieva et al., 2022; Madsen et al., 2023), and tetra-VH IgG (Ljungars et al., 2020; Misson Mindrebo et al., 2023) (Figure 2). A strong practical advantage of symmetric bsAbs resides in the relatively simple antibody assembly where only one or two different polypeptide chains need to be expressed. This means there is less optimization of plasmid transfection ratios in initial screens compared to co-expression of three to four chains for asymmetric bsAbs. The HC_2_LC_2_ format also greatly reduces the risk of misassembly of antibody chains and thus also simplifies the purification scheme compared to asymmetric bsAbs where additional polishing steps are often needed to remove impurities formed through incorrect assembly of antibody chains (Li, 2019). The HC_2_LC_2_ format, however, also limits the flexibility in valencies as the antigen-binding domains will always appear in pairs. This limits the application to antigens where monovalent targeting is required to prevent undesired crosslinking, such as CD3 (Lee et al., 2019). The choice of a suitable exogenous antigen-binding domain is an important consideration when designing symmetric bsAbs. Traditionally, scFvs have often been used because they can be readily derived from Fv domains of conventional antibodies. Smaller sdAbs are, however, gaining increasing attention as attractive fusion partners because they are naturally small and monomeric and thus not prone to undesired self-assembly and aggregation, which is often seen for scFvs (Cao et al., 2018; Andrade et al., 2019).

Glycine-serine linkers of 10–25 amino acids are commonly used for fusion of exogenous antigen-binding domains as these exhibit favorable flexibility and stability in aqueous solutions (Chen et al., 2013). Other bsAb constructs have utilized linkers derived from natural antibody linker regions such as the antibody hinge region (Kuo et al., 2012) or the flexible link connecting Fv and CH1/Cκ (Wu et al., 2007; Wu et al., 2009). The choice of an appropriate linker is important to ensure proper spacing and display of the antigen-binding domains (Wu et al., 2009; Madsen et al., 2023). One study found linker lengths to affect both antigen-binding and stability of DVD-Ig molecules (Wu et al., 2009).

The highly modular nature of antibodies means that the exogenous antigen-binding domains can be fused both within or at the ends of polypeptide chains of the scaffold, thus enabling formation of structurally diverse bsAbs that can be tailored to fit the purpose (DiGiandomenico et al., 2014; Swope et al., 2020). While fusions of these exogenous antigen-binding fragments are often made onto full length IgG molecules they can also be fused directly to Fc domains to keep the constructs smaller than full length IgG molecules while still including the Fc region (Asano et al., 2020). In addition to the fusion of independent antigen-binding domains onto IgG scaffolds, other strategies have been employed to develop symmetric IgG-like bsAbs. One example is formation of tetra-VH IgGs by separating out distinct binding specificities onto each variable domain of the Fv by replacing VH and VL with independent sdAbs (Ljungars et al., 2020). It has also been possible to spatially segregate the 6 complementarity-determining regions (CDRs) of a single Fab domain into a VH paratope (CDRH1, CDRL2 and CDRH3) and a VL paratope (CDRL1, CDRH2 and CDRL3) (Beckmann et al., 2021). Combining these individual paratopes allowed formation of a single bispecific Fab domain dubbed DutaFab.

## 5 Asymmetric bispecifics

The molecular geometry of asymmetric bsAbs makes them more challenging to design and produce compared to symmetric bsAbs mainly due to formation of antibody-related impurities formed through co-expression of the different antibody chains. Despite these practical issues, asymmetric bsAbs are still the major type of bsAb entering into clinical development (Labrijn et al., 2019). A notable advantage of asymmetric bsAbs is the close adherence to the native molecular geometry of IgG to harness the favorable drug-like qualities of this molecule.

The asymmetric architecture allows high flexibility in valencies, e.g., to form monovalent specificities (1 + 1) (Kienast et al., 2013; Zhang et al., 2015), which cannot be obtained for symmetric bsAbs. Further, formation of asymmetric bsAbs with high structural resemblance to IgG molecules is believed to reduce the risk of immunogenicity liabilities. It should, however, be noted that asymmetric bsAbs can also be formed to deviate from the strictly bivalent Y-shaped IgG geometry to form, e.g., trivalent 2 + 1 bsAbs (Metz et al., 2012; Bacac et al., 2016a; Bacac et al., 2016b).

### 5.1 HC:HC pairing

The main issue associated with asymmetric bsAbs is mispairing of polypeptide chains leading to product-related impurities. Many asymmetric bsAbs rely on heterodimerization of two different HC, thus spiking interest in HC steering platforms for promoting HC heterodimerization. Numerous HC steering platforms have been developed (Liu et al., 2017), of which the majority is industry-originated, and functions by creating complementary interfaces in the CH3 domains (Ridgway et al., 1996; Atwell et al., 1997; Merchant et al., 1998; Davis et al., 2010; Gunasekaran et al., 2010; Moore et al., 2011a; Choi et al., 2013; Labrijn et al., 2013; Von Kreudenstein et al., 2013; Choi et al., 2015a; Choi et al., 2015b; Leaver-Fay et al., 2016; De Nardis et al., 2017; Skegro et al., 2017; Estes et al., 2021), which are key in governing the antibody structural integrity (Feige et al., 2010; Bertz et al., 2013). The mutations in the structurally important CH3 domain are therefore also known to often affect the bsAb thermal stability (Pomarici et al., 2022). While the detailed design principles of these CH3 steering platforms are thoroughly reviewed elsewhere (Ha et al., 2016; Brinkmann and Kontermann, 2017), it should be noted that the platforms seek to introduce structurally complementary mutations that favors HC heterodimerization while disfavoring formation of HC homodimers. The first reported, and most widely used, platform is the knob-into-hole (KiH) which introduces a large bulky tryptophan in one HC and smaller sterically complementary residues in the other HC (Atwell et al., 1997; Merchant et al., 1998). The KiH strategy is widely implemented because it is highly effective in suppressing HC homodimers (except for trace amounts of hole-hole homodimers (Chen et al., 2019; Tang et al., 2020)) and because its patent has expired. The KiH thus provides relatively easy and free access to construction of asymmetric bsAbs in a field that is otherwise highly competitive and industry-dominated as illustrated by several other patent-protected platforms (Godar et al., 2018).

When engineering HC for heterodimerization it should be realized that the relative abundance of HC heterodimer should be considered an equilibrium rather than a fixed amount. This equilibrium is affected by several factors such as the relative plasmids- and expression levels, which is typically controlled through optimizing plasmid transfection ratios (Ding et al., 2021; Ong et al., 2022). This can be time consuming especially if expressing many different bsAbs. As an example, we recently reported production of an asymmetric IgG/sdAb-Fc construct where a skewed HC plasmid transfection ratio yielded the most equal expression of the two HC chains (Madsen et al., 2022), thus suggesting the two HCs did not co-express equally well. Other studies have similarly found that the nature of the variable domains affect HC heterodimerization (Stutz and Blein, 2020; Estes et al., 2021). The CH1 domain plays an important role in the assembly and transport of IgG molecules (Hendershot et al., 1987; Feige et al., 2009), which can help explain changes in expression behavior of HCs where the Fab domain has been replaced with fragments such as sdAbs or scFvs. Interestingly, improved assembly of heterodimeric κλ bsAbs has also been obtained through codon de-optimization of the high-expressing λ chain to balance the relatively low-expressing κ thus actually increasing the yield of the target bsAb (Magistrelli et al., 2017).

### 5.2 HC:LC pairing

In addition to HC heterodimerization, strategies also exist for ensuring correct HC:LC pairing. Selection of bsAbs with common LC eliminates the risk of HC:LC mispairing (Merchant et al., 1998; Sampei et al., 2013) although identification of common LC bsAbs can be time consuming and restrictive in terms of sequence diversity (Sampei et al., 2013; Shiraiwa et al., 2019; Ching et al., 2021), which may complicate the search for target-specific and high-affinity binders. The common LC approach further has the advantage that only three polypeptide chains need to be expressed thus easing manufacturability to a certain extent. Analogously, bsAbs with common HC can also be discovered to avoid HC mispairing (Fischer et al., 2015). Other approaches have focused on rational design of Fab steering that prevents HC:LC mispairing during co-expression of the different polypeptide chains, including Crossmab (Schaefer et al., 2011), orthogonal Fab interfaces (Lewis et al., 2014; Liu et al., 2015; Dillon et al., 2017; Froning et al., 2017; Koga et al., 2023), swapping of CH1/CL domains (Wu et al., 2015; Wozniak-Knopp et al., 2018), engineering of native interchain disulfide bonds (Mazor et al., 2015b), and grafting of IgE-derived heterodimerization domains (Kühl et al., 2022). The use of two different HC and LC allows flexible pairing of VH and VL domains and thus unrestricted access to antibody diversification when searching for target-specific bsAbs. On the other hand, balanced co-expression of all 4 polypeptide chains can be challenging thus potentially requiring additional efforts in vector design and production clone generation as well as added pressure on downstream processes. Proper HC:LC pairing can also be obtained through careful post-expression assembly where each antibody half is expressed individually and subsequently assembled to the final bsAb construct. This strategy, however, introduces additional manufacturing steps such as clone generation and manufacturing of 2 components before combination adding further challenging processing steps including sufficient reduction and oxidation of hinge disulfides (Strop et al., 2012; Labrijn et al., 2013; Spiess et al., 2013; Labrijn et al., 2014). Oher strategies include replacing one of the Fab arms with a single-chain Fab (scFab) domain so the bsAb consists of only 3 polypeptide chains and where the flexible linker of the scFab promotes proper pairing of VH/CH1 and VL/CL (Schanzer et al., 2014).

Interestingly, some Fab domains appear to exhibit inherent preferential cognate HC:LC pairing whereas other Fabs exhibit a universally more promiscuous HC:LC pairing (Dillon et al., 2017; Joshi et al., 2019; Gong et al., 2021). The determinants of pairing are mainly located in the CDRs, and such insights might be applicable in selecting compatible HC:LC pairs or identifying common LC. The issue of HC:LC mispairing can also be avoided by simply replacing one or both Fabs with antibody-fragments, such as scFv fragments or sdAbs, to ensure that the bsAb contains a single LC at most thus avoiding HC:LC mispairing (Huang et al., 2020; Ne et al., 2020; Madsen et al., 2022). The importance of proper chain pairing of bsAbs has also spiked interest in advanced analytics and efficient downstream purification processes for accurately removing and quantifying mispaired species with high throughput (Schachner et al., 2016; Yin et al., 2016; Yan et al., 2019; Ziegengeist et al., 2023).

### 5.3 Alternative chain steering

In addition to the assembly methods described above, other strategies more heavily relying on chemical processing have also been explored. One example is expression of an IgG-like bsAb consisting of 4 different polypeptide chains expressed as a single construct where the antibody chains had been connected by linkers that steered chain assembly and could be removed post-expression (Dimasi et al., 2017). BsAb chain association has also been controlled by appending leucine zippers (Wranik et al., 2012) and full-length IgG molecules have been combined through SpyTag/SpyCatcher system (Mei et al., 2022) or using click chemistry (Wagner et al., 2014).

## 6 Molecular geometries

BsAbs are more than just the sum of their parts and selection of an optimal molecular architecture, is an important design consideration to obtain the desired functionality. This means that bsAbs that are constructed from the same molecular building blocks (and thus sharing the same total amino acid content) only differing in molecular geometry can exhibit varying activity (Dickopf et al., 2020; Madsen et al., 2023). The dual targeting is often complex because the bsAb configuration must account for both internal and external restraints to obtain the desired therapeutic functionality. Internal restraints are imposed by the molecular geometry in itself such as steric hindrance between the binding domains (Do et al., 2020; Madsen et al., 2023). One study examining a comprehensive set of symmetric sdAb-IgG bsAbs including mirroring the specificities found that the binding affinity of the antigen-binding domains was affected by inter-domain steric hindrance arising from the molecular geometry and that this effect was more pronounced when the sdAb was linked to the LC as compared to the HC (Madsen et al., 2023). Such internal restraints might be alleviated through engineering of the configuration, e.g., by extending linkers to increase the intramolecular flexibility and distance between target binding (Wu et al., 2007). External restraints, on the other hand, are imposed by the spatial organization of the target environment where the bsAb must adopt a specific conformation to achieve the desired spatiotemporal target engagement. Such external restraints are especially evident for bsAbs with (inter-)cellular activity (Croasdale et al., 2012; Kühl et al., 2023) due to the inherently complex nature of spatial cell surface receptor organization (Bethani et al., 2010). As an example, the immunological synapse required for T cell activation is dependent on the spacing of the T cell antigen receptor and the peptide-MHC ligand (Choudhuri et al., 2005). Given that artificial immunological synapses, established through T cell redirecting bsAbs, have shown high phenotypical similarity to natural immunological synapses (Offner et al., 2006) it is worthwhile considering the natural spacing restraints when designing these T cell redirecting bsAbs. In another example, FynomAbs demonstrated higher cytotoxic activity when the Fynomer (small binding protein derived from the SH3 domain from Fyn kinase) was attached N-terminally as opposed to C-terminally thus further illustrating how the geometry can be tailored for optimal interparatopic positioning (Wuellner et al., 2015). Small fragment-based configurations might also be applicable in cases where short interparatopic distances are favorable. The MOA can likewise be influenced through judicious design of the molecular configuration and interparatopic spacing of antigen-binding domains. This has been shown for T cell engaging scFv-IgG bsAbs where shorter interparatopic spacing obtained through fusion of the scFv C-terminally on the LC proved superior anti-tumor effect both *in vitro* and *in vivo* (Santich et al., 2020). Similarly, fragment-based DART molecules targeting CD19 and CD3 have shown superior cell lysis compared to a tandem scFv composed the same binding domains, thus indicating that the more compact configuration of DART molecules are favorable for maintaining cell-cell contacts (Moore et al., 2011b).

The importance of spatial receptor organization has spiked interest in structural analyses of receptor:antibody complexes aiming to uncover underlying mechanisms of effective antibody binding (Lee et al., 2016; Li et al., 2017; Chin et al., 2018). Ideally, such findings will aid the design of improved antibody therapeutics. The importance of the interparatopic distance is not only important in inter-cellular targeting but also for bsAbs where both epitopes are located on the same target cell (DiGiandomenico et al., 2014). For example, the ability of biparatopic anti-HER2 bsAbs to effectively trap HER2 in inactive crosslinked confirmations were highly dependent on the molecular geometry (Kast et al., 2021).

Given the influence of the bsAb configuration, the most potent and effective bsAb cannot be ascertained from analysis of parental monospecific antibodies alone. Efforts are thus made to develop functional screening strategies that goes beyond assessment of parental antibody binding alone and enables screening of combinatorial bsAb panels to also evaluate optimal molecular geometry and combinations of antigen-binding domains (Geuijen et al., 2018; Dengl et al., 2020; Segaliny et al., 2023).

Furthermore, it is not only the molecular geometry that is affecting the bsAb potency but also the relative orientation of the specificities. As an example, significantly reduced HER2 binding was observed when fusing anti-HER2 scFv to an anti-PD1 IgG scaffold compared to reverse orientation, i.e., fusion of the anti-PD1 scFv to the anti-HER2 IgG (Guling et al., 2022). Similar effects from relative orientation have also been shown for fragment-based bsAbs (de Bruin et al., 2018; Andres et al., 2019). In the design of bsAbs, it is not only the molecular configuration and the valencies that must be optimized to achieve the desired MOA but also the relative binding affinities between the different antigen-binding arms. There might thus be a need evaluate combinations of affinity variants to identify the optimal relationship. The importance of balancing the binding affinities has raised interest in mechanistic modelling for understanding the affinity interplay to allow informed bsAb design (Rhod et al., 2016; Kareva et al., 2018; Kareva et al., 2021). This affinity tuning has proven especially important for T cell engaging bsAbs, targeting CD3 and a tumor-associated antigen, and where the relative binding affinities have been shown to affect both efficacy and selectivity (Haber et al., 2021).

## 7 Developability considerations

In addition to efficient and specific antigen-binding, successful therapeutic bsAbs must possess favorable drug-like qualities, such as high expression, good biophysical stability, low self-association, and aggregation as well as excellent solubility. These traits are commonly known as the developability profile and screening for developability is typically done early in the drug development process to avoid investing in antibodies that are unlikely to succeed as clinical candidates (Jain et al., 2017; Raybould et al., 2019). Extensive work has been aimed at developing *in silico* predictive tools and high-throughput assays for early screening of candidate developability liabilities (Bailly et al., 2020; Mieczkowski et al., 2023; Svilenov et al., 2023). The methods were, however, primarily developed for conventional monoclonal antibodies and extra attention might therefore be required for bsAbs because the engineering strategies used for constructing the bsAb also risk introducing unexpected liabilities. As an example, we recently showed that fusion of sdAbs onto IgG scaffolds cause changes in the expression yields and biophysical stability and that these changes were dependent on the molecular geometry, the sdAb fusion site on the IgG scaffold, and the number of domains fused (Madsen et al., 2023). This study thus highlights the importance of an optimal molecular geometry and that the bsAb developability profile cannot be ascertained from analysis of the individual building blocks or the parental antibodies alone.

Other examples of developability liabilities potentially introduced by a bsAb configuration include fragmentation, aggregation propensity as well as reduction and re-oxidation of engineered disulfides (Cao et al., 2018; Andrade et al., 2019; Swope et al., 2020; Cramer et al., 2023). Molecular geometry is thus an important design consideration also with respect to chemical and biophysical stability. Further, the molecular format has also been shown to influence the pharmacokinetics of the bsAb (Datta-Mannan et al., 2016; Datta-Mannan et al., 2021) and efforts have been made to establish *in vitro* assays for predicting bsAb pharmacokinetics (Müller et al., 2023).

The general developability properties of bsAbs are not very well characterized and compared across the large variation of format and geometries available potentially due to the sheer number of bsAb formats (Brinkmann and Kontermann, 2017), making it difficult to generalize on bsAb developability. While larger systematic studies would be advantageous to establish more comparative common rules and developability properties of various molecular formats and geometries the current approach often aims to identify bsAbs with favorable developability profiles within certain intellectual property platform spaces through high-throughput screening of combinatorial libraries and/or through rational multiparameter optimization (Sampei et al., 2013; Dengl et al., 2020; Furtmann et al., 2021; Root et al., 2021).

While some of the very high-throughput screening pipelines are dependent on advanced and automated instrumentation that is not readily available in most research labs, combinatorial bsAb panels for screening can also be generated through bioconjugation of individually expressed antibody components that are assembled to the final bsAb post-expression (Labrijn et al., 2014; Dengl et al., 2020; Hofmann et al., 2020). Practically, this allows construction of large bsAb panels without having to go through tedious production of each individual bsAb thus enabling screening of combinatorial binder formats.

Rational improvement of bsAb developability typically entails targeted optimization of the problematic fragment(s). Examples include engineering fragments for increased thermal stability (Miller et al., 2010; Von Kreudenstein et al., 2013; Boucher et al., 2023), solubility (Sampei et al., 2013), chemical stability (Sampei et al., 2013) and aggregation (Miller et al., 2010; Boucher et al., 2023). It is, however, important that optimized domains are subsequently re-evaluated in the context of the full length bsAb. Requirements for drug-like qualities can also be included already in the discovery, e.g., by including selective pressure for drug-like qualities in the screening process (Jespers et al., 2004).

## 8 Conclusion

Bispecific antibodies (bsAbs) represent a highly promising class of therapeutic modalities, demonstrating significant potential in various medical applications. The success of these molecules is highly dependent on efficient design and construction, which requires careful consideration to ensure optimal balance between therapeutic potency and favorable physicochemical properties. As previously demonstrated, the intricate interplay between the function and performance of bsAbs is intricately tied to their structural configuration. As discussed in this work, considerable efforts are directed towards the engineering of bsAbs with dual binding activity, while concurrently addressing the imperative need for developability profiles that align with, or even surpass, those of conventional monospecific antibodies. The comprehensive insights presented herein underscore the complexity of bsAb engineering, emphasizing the importance of strategic design to harness their full therapeutic potential. Notably, our exploration delves into the nuances of engineering strategies and practical considerations, shedding light on the challenges and opportunities inherent in efficient bsAb design. By dissecting the intricacies of these design principles, we contribute to the continued advancement of bsAbs as versatile and effective therapeutic agents, providing a roadmap for future research and development of improved bsAb therapeutics.
